# Investigating workload and usability of remote magnetic navigation for catheter ablation

**DOI:** 10.1007/s11548-025-03558-z

**Published:** 2025-12-15

**Authors:** Florian Heemeyer, Leonardo E. Guido Lopez, Miguel E. Jáuregui Abularach, Beatriz Sanz Verdejo, Quentin Boehler, Oliver Brinkmann, José L. Merino, Bradley J. Nelson

**Affiliations:** 1 Multi-Scale Robotics Lab, ETH Zurich, Tannenstr. 3, 8092 Zurich, Switzerland; 2 Arrhythmia and Robotic Electrophysiology Unit, La Paz University Hospital, Paseo de La Castellana 261, 10587 Madrid, Spain

**Keywords:** Remote magnetic navigation, Electromagnetic navigation system, Catheter ablation, Robotic surgery, Telesurgery, Workload, Usability

## Abstract

**Purpose:**

Robotic systems for catheter ablation have been in clinical use for many years. While their impact on the clinical outcome and procedure times is well studied, aspects like usability and operator workload have received limited attention in the literature. Reduced workload and stress levels benefit the operator’s mental and physical health, and can also lower the risk of errors and ultimately improve patient safety. The aim of this study is to investigate the workload and usability of remote magnetic navigation compared to conventional manual navigation.

**Methods:**

We performed a user study with eight electrophysiologists. Each participant performed identical in-vitro navigation tasks replicating those found in pulmonary vein isolation using both manual and magnetic navigation. Magnetic navigation experiments were performed using the Navion, a mobile electromagnetic navigation system.

**Results:**

Magnetic navigation significantly improved usability (*p* < 0.02) and workload (*p* < 0.01) compared to manual navigation, measured using the System Usability Scale (magnetic: 85.6 ± 9.3 vs. manual: 75.0 ± 17.8) and NASA Task Load Index (magnetic: 72.4 ± 13.5 vs. manual: 45.8 ± 16.7). Additionally, task completion times were shorter (*p* < 0.01) with magnetic navigation (284.6 ± 80.7 s) compared to manual navigation (411.0 ± 123.7 s).

**Conclusion:**

The findings of this study suggest that remote magnetic navigation using the Navion significantly improves operator experiences in terms of workload and usability, reinforcing the case for wider adoption of well-designed robotic systems in cardiac electrophysiology labs.

**Supplementary Information:**

The online version contains supplementary material available at 10.1007/s11548-025-03558-z.

## Introduction

Cardiac arrhythmias are common conditions characterized by abnormal heart rhythms and an increased risk of thromboembolic events. Catheter ablation is a guideline-recommended therapy for a wide range of cardiac arrhythmias [1, 2], targeting and selectively destroying abnormal myocardial tissue responsible for aberrant electrical conduction. Despite its proven effectiveness, catheter ablation is technically demanding and time-intensive when performed manually, making it well-suited for assistive robotic technologies.

Robotic systems for catheter ablation procedures have been introduced in recent years with the intention of improving performance and usability. Notable commercial systems include the Genesis and Niobe systems by Stereotaxis, the Sensei X system by Hansen Medical [3], and the Amigo system by Catheter Precision Inc. [4]. In addition, the development of robotic systems and ablation catheters remains an active field of research [5–8]. Robotic assistance is associated with reduced X-ray exposure [9], swift back-tracking [10], and improved performance in reaching difficult anatomical targets [10]. Magnetically actuated systems, in particular, have shown better catheter stability [10, 11] and improved safety [11]. Robotic systems can also improve operational factors, such as operator workload and usability, but the current literature lacks systematic investigation of these aspects.

Despite receiving limited attention in existing literature, workload and usability are important factors. Reduced stress levels and physical demand can have a positive effect on the operator’s overall well-being [12] and can help prevent burnout, a common issue among surgeons [13]. Surgeon burnout is estimated to cause approximately $4.6 billion in economic losses each year in the United States [14]. Additionally, high stress levels are associated with higher risks of malpractice [12]. Thus, effective stress reduction could reduce the risk of stress-related errors. A reduced workload could also decrease the risk of musculoskeletal injuries due to extended periods in non-neutral postures during the procedure [15].

**Fig. 1:**
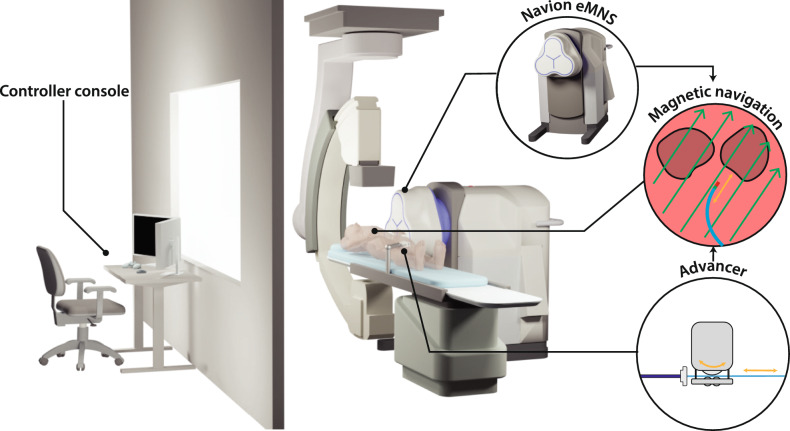
Catheter ablation with the Navion. The system consists of a controller console and a robotic system. The console displays data such as the cardiac map, electrograms, and fluoroscopy images for feedback. A controller allows the operator to send commands to the robotic system. The Navion includes the eMNS and an advancer. The eMNS generates a magnetic field (green arrows) to steer the tip of the magnetic catheter, while the advancer inserts and retracts the catheter (orange arrows). The controller console can be located in an adjacent room or operated remotely in a telesurgery setting

In this work, we systematically evaluate the performance of remote magnetic catheter navigation compared to conventional manual catheter navigation, focusing on usability and workload. We investigate the Navion [16], a mobile electromagnetic navigation system (eMNS) consisting of three electromagnets. Figure 1 illustrates the concept of catheter ablation with the Navion, as well as a potential room configuration in which the Navion is positioned laterally beside the patient and the fluoroscope is placed at the head position. The user study presented in this work leverages the evaluation platform presented in [17, 18]. While that earlier work detailed the technical aspects of the evaluation platform, this work focuses on the user study and its results. The magnetic navigation experiments were conducted in a telesurgery setting, with electrophysiologists in Madrid remotely operating the robotic system located in Zurich.

## Methods

### Navigation methods

**Fig. 2:**
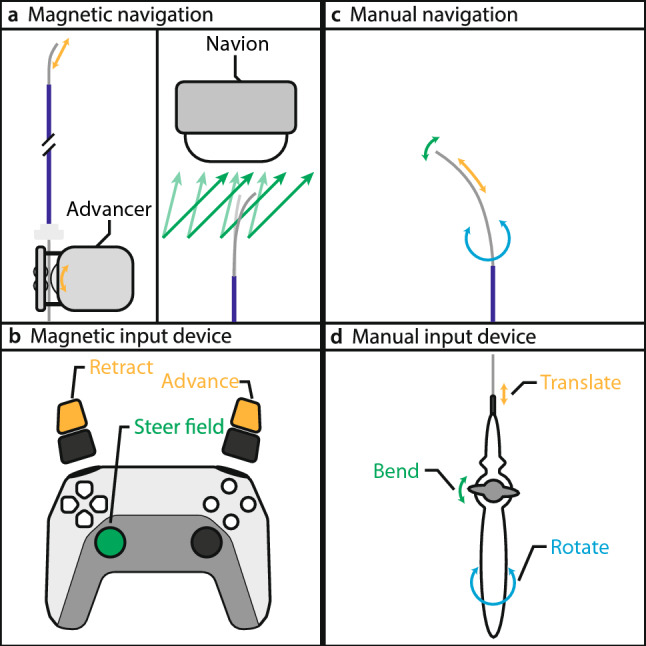
Navigation methods compared in this study. **a** Magnetic navigation steers a magnetic catheter by controlling the external magnetic field and translating the catheter through a mechanical advancer unit. **b** Control inputs are given through a handheld controller. **c** Manual catheter navigation involves bending, translating, and rotating the catheter tip. **d** These movements are achieved through a handle at the catheter base that mechanically transmits the operator's inputs to the tip

The goal of this study was to assess the performance of remote magnetic navigation in catheter ablation, focusing on the aspects of usability and workload. Each participant performed identical experiments using two navigation methods: first, conventional manual catheter navigation, followed by remote magnetic navigation. The two navigation methods are illustrated in Fig. 2.

For the magnetic navigation experiments, we used the Navion, a human-scale eMNS capable of generating magnetic fields of up to 25 mT in any direction within its workspace [16]. The system also enables hybrid usage of operating rooms, since it is mobile and can easily be rolled in and out as needed. It can generate magnetic fields in arbitrary directions and thereby control a variety of magnetic instruments, including ablation catheters. When a magnetic catheter is placed within an external magnetic field, a magnetic torque is exerted on the catheter that acts to align it with the externally applied field. The base of the magnetic catheter is clamped into an advancer unit that advances and retracts the catheter. The operator controls the magnetic field and the advancer unit using a PlayStation 5 controller (Sony, Tokyo, Japan). We chose this controller because it is widely available, ergonomically optimized through its extensive use in the gaming industry, and provides the necessary control inputs for magnetic field steering and catheter translation. For the magnetic navigation experiments, we used the Thermocool RMT Catheter (Johnson & Johnson, New Brunswick, NJ, USA).

Manual catheter navigation is currently the standard-of-care method to steer ablation catheters. The operator stands beside the operating table and manipulates the catheter base, which is mechanically coupled to the catheter tip. The operator navigates the catheter tip to the desired target by translating, rotating, or turning the knob on the catheter handle. For the manual navigation experiments, we used the TactiFlex Ablation Catheter (Abbott Laboratories, Abbott Park, IL, USA).

### Experimental setup

**Fig. 3:**
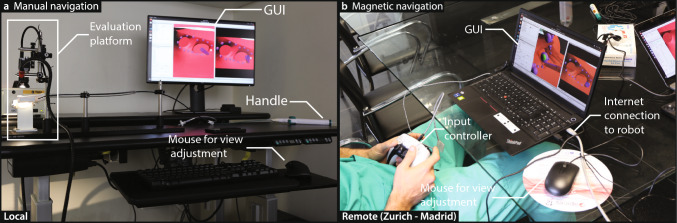
Experimental setups. **a** Manual navigation: The participant stands at the setup and navigated the catheter tip using the catheter handle. **b** Magnetic navigation: The participant controls the catheter tip remotely using a controller. The controller console was connected to the robotic system via the internet. In both setups, participants navigated the catheter based on the visual feedback provided by the GUI, which allowed view adjustments via a mouse

The experimental setups for manual and magnetic navigation are shown in Fig. 3. We used identical evaluation platforms designed to simulate catheter ablation navigation for both navigation methods. Each platform consisted of a 3D-printed heart model, a camera, and a light source. A sheath was placed inside the left atrium of the model, and the respective catheter was advanced through the sheath such that its tip was located inside the left atrium.

**Fig. 4:**
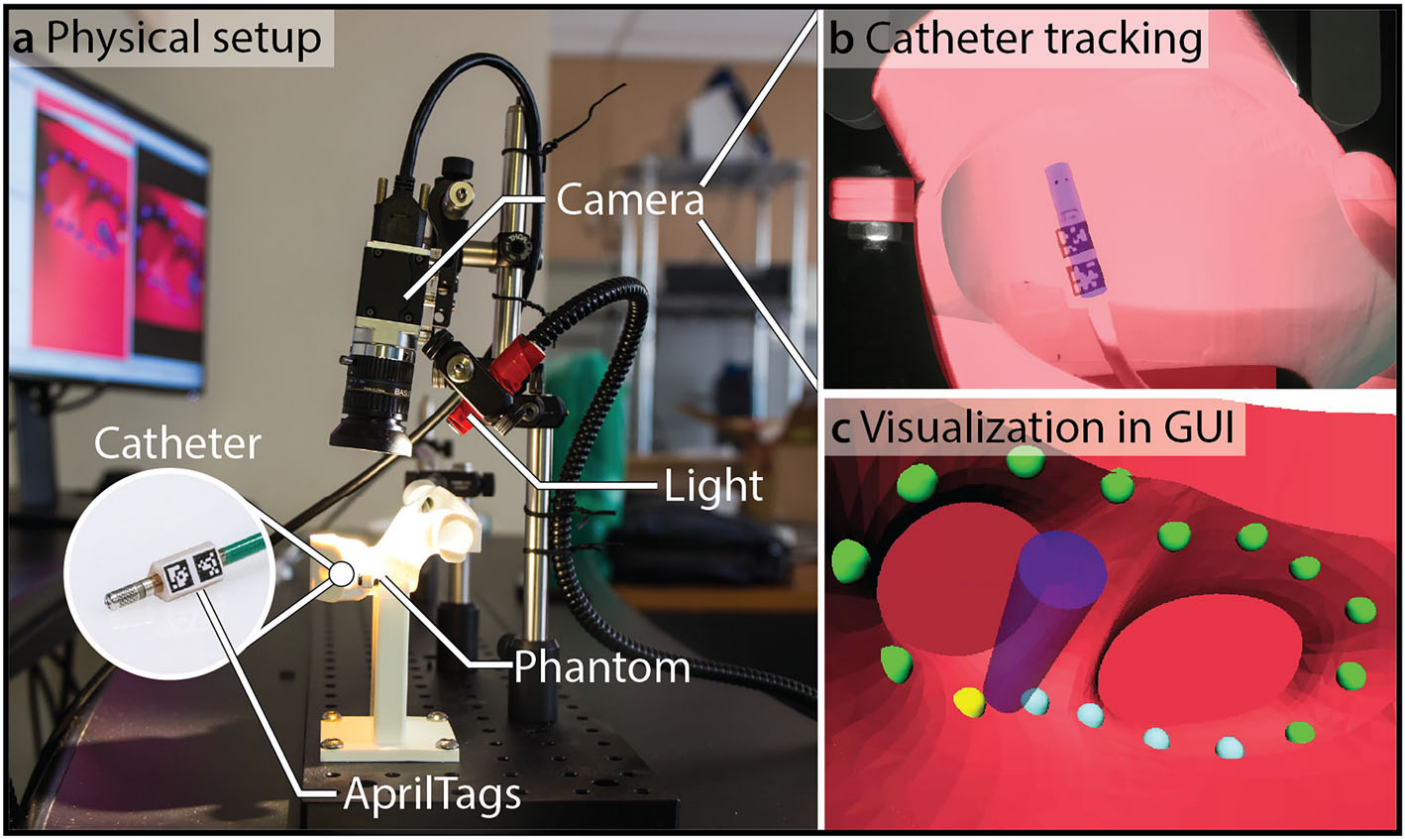
Catheter tracking. **a** Physical setup showing the 3D-printed heart phantom, camera, light source, and catheter equipped with AprilTag markers near its tip inside the phantom. **b** Example camera view of the catheter tip with visible AprilTags inside the phantom, enabling real-time pose estimation using a single static camera. **c** Visualization in the GUI, where the tracked catheter (blue) and predefined navigation targets are displayed. Because the spatial relationship between the camera and the phantom was known, the catheter pose could be computed and visualized within the GUI, allowing interactive perspective adjustments similar to those in clinical mapping systems

The catheter tracking setup is illustrated in Fig. 4. The catheter’s pose was determined using an AprilTag-based optical tracking system, which provided full pose estimation from a single fixed camera. AprilTags are visual fiducial markers [19] that can be localized in an image, allowing accurate real-time tracking of the catheter tip. The computed catheter pose was visualized in real-time in a graphical user interface (GUI) that displayed the phantom, catheter, and predefined navigation targets. Participants navigated the catheter toward these targets using the feedback provided in the GUI, which offered both a fixed and an adjustable view of the interventional scene. Further details on the evaluation platform can be found in [17, 18].

The participants performed the manual navigation experiments locally, standing at the setup as they would in a conventional procedure. For these experiments, the manual catheter was inserted into the sheath, and the participants steered the tip using the catheter handle.

The magnetic navigation experiments were performed remotely. The Navion was located at ETH Zurich (Zurich, Switzerland), with the evaluation platform placed on an operating table within the system’s workspace to enable remote control of the magnetic catheter. The experimental setup was oriented such that the opening of the left pulmonary veins faced the Navion, creating a magnetic gradient that pulled the catheter toward the veins. The controller console was located at the La Paz University Hospital (Madrid, Spain). An internet connection between the robotic system and the controller console was established, allowing the operator to control the magnetic field and the advancer unit of the system from a distance, a mode of operation often referred to as telesurgery [20]. Both sites were connected via high-speed wired internet with sufficient bandwidth, resulting in a round-trip latency of approximately 41 ± 1 ms. During the remote magnetic experiments conducted from Madrid, an engineer was present on-site in Zurich to monitor the robotic system.

Supplementary videos S1 and S2 demonstrate magnetic and manual navigation experiments, respectively.

### Study protocol

**Fig. 5:**
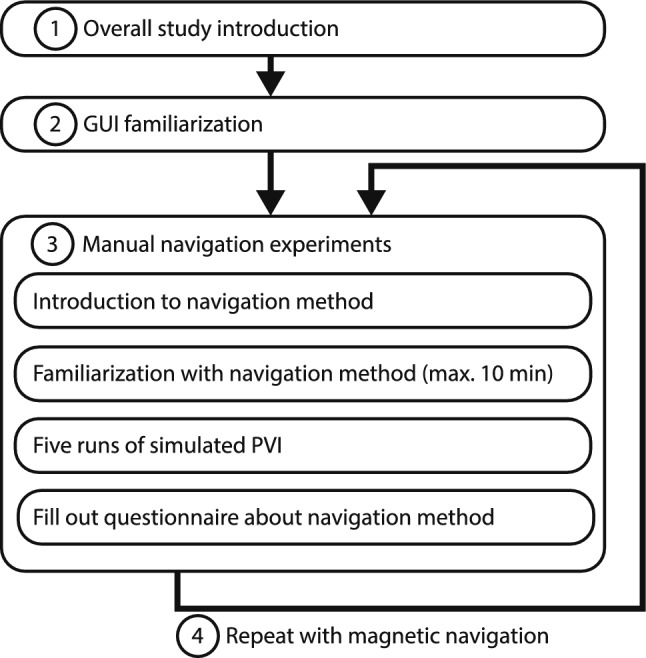
Study protocol. Each participant completed the study following a structured protocol, which included four main steps: a general study introduction, GUI familiarization, manual navigation experiments, and magnetic navigation experiments

The study participants were eight electrophysiologists. They were informed about the data that would be collected and were given the opportunity to ask questions. Informed consent was obtained from all participants prior to the study. The study protocol is shown in Fig. 5. Each participant performed two sets of five navigation runs: first using manual navigation and then repeating the same tasks with remote magnetic navigation. Each run exceeding 20 min was stopped and considered unsuccessful. The catheter position was reset after each run. Participants were instructed not to look at the physical model and navigate exclusively using the feedback from the GUI. To capture the performance of the navigation methods, participants filled out questionnaires after completing the five runs. The questionnaires were a System Usability Scale (SUS) assessment and a NASA Task Load Index (TLX) evaluation.

**Fig. 6:**
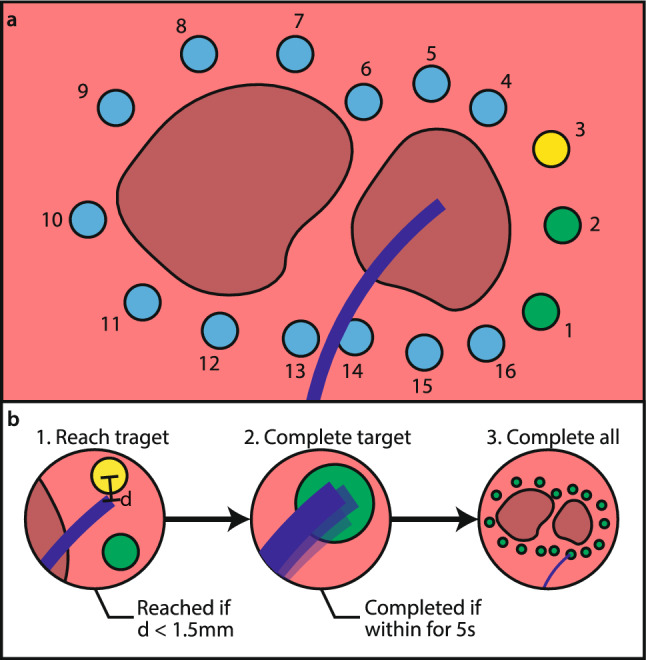
Navigation task. **a** Predefined ablation targets are equally spaced at 6 mm around the pulmonary veins. The order in which the targets had to be completed is indicated by the numbers, and was identical for each run. **b** A target was considered reached if the catheter tip was within 1.5 mm of the next target's center, and completed if it remained within the target for five consecutive seconds

The navigation task, shown in Fig. 6, simulated a typical navigation task as found in pulmonary vein isolation. The objective in each run was to navigate the catheter tip to all 16 targets and simulate energy delivery by holding the catheter in a steady position for five consecutive seconds at each target. A five-second duration of simulated energy delivery was chosen to allow participants to complete multiple runs within a reasonable time limit and still introduce some degree of physical fatigue. The GUI displayed the catheter’s pose inside the model along with the position of all targets. The targets were color-coded in three colors: green for completed targets, yellow for the next target, and blue for remaining targets. For color-blind participants, the colors were adjusted to purple, white, and orange.

Participants had to navigate the catheter to the next target, which was considered to be reached if the distance, *d*, between the catheter tip and the center of the defined target was below 1.5 mm. Once a target was reached, the catheter had to be maintained within the target for five consecutive seconds before the participant could move to the next target. If the catheter could not be held within the target for the full five seconds, the timer was reset to zero, and the target had to be reached again. A run was considered to be completed if all 16 targets were completed in under 20 min.

To compare the performance of the two navigation methods, we assessed usability and workload using standardized questionnaires filled out by each participant immediately after finishing a navigation method. Usability was measured using the SUS Score for each method. The SUS captures various aspects of a system's usability, including effectiveness, efficiency, and user satisfaction. The SUS questionnaire consists of ten items, each rated on a five-point Likert scale, ranging from 1 (strongly disagree) to 5 (strongly agree). The total SUS score, ${SUS}_{total}\in \left[\mathrm{0,100}\right]$, can be calculated as follows:

$$ \begin{aligned} {\mathrm{SUS}}_{{{\mathrm{total}}}}  = \left( {\sum\limits_{{i \in \{ {\mathrm{1,3}},{\mathrm{5,7}},9\} }} {\left( {{\mathrm{SUS}}_{i}  - 1} \right) + } \sum\limits_{{j \in \{ {\mathrm{2,4}},{\mathrm{6,8}},10\} }} {\left( {5 - {\mathrm{SUS}}_{j} } \right)} } \right) \times 2.5 \end{aligned}$$

Workload was assessed using the NASA TLX. The NASA TLX questionnaire consists of six items related to various aspects of workload: mental demand, physical demand, temporal demand, performance, effort, and frustration. Participants rated each question on a five-point Likert scale from 1 (very low) to 5 (very high) with the exception of the performance-related question. The performance scale ranged from 1 (success) to 5 (failure). The overall NASA TLX score, ${\mathrm{TLX}}_{\mathrm{total}}\in \left[\mathrm{0,100}\right]$, is calculated as follows:

$$\mathrm{TL}{X}_{\mathrm{total}}={\sum }_{i=1}^{6}\left(5-{\mathrm{TLX}}_{i}\right)\times \frac{25}{6}$$

Finally, the completion times of all runs were recorded.

### Statistical methods

Data collected during the study were analyzed using statistical methods for a paired experimental design. Since each participant performed the same experiments using both navigation methods, we created paired datasets for each metric and questionnaire item. One-sided paired t tests were performed for each metric and questionnaire item to evaluate whether magnetic navigation significantly outperformed manual navigation. All comparisons were based on paired data, and the critical significance level was set to 0.05.

## Results

### Usability

**Fig. 7:**
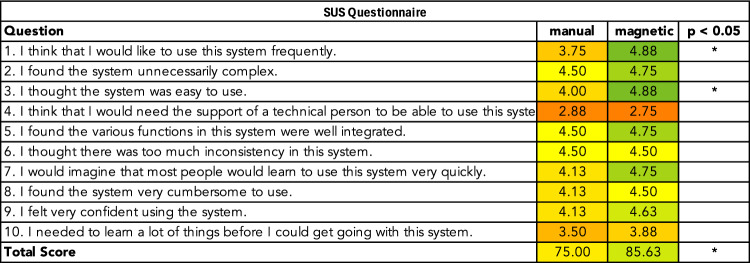
SUS ratings. Each row represents the average rating for the corresponding item on a five-point Likert scale, with 1 (worst) and 5 (best), for manual and magnetic navigation. For improved readability, scores were inverted for items for which lower values are more desirable

The average ratings for each item of the SUS questionnaire are shown in Fig. 7. Magnetic navigation significantly improved the overall SUS Score from 75.0 ± 17.83 for manual navigation to 85.6 ± 9.33 for magnetic navigation. In particular, it showed statistically significant improvements in items 1 and 3.

### Workload

**Fig. 8:**
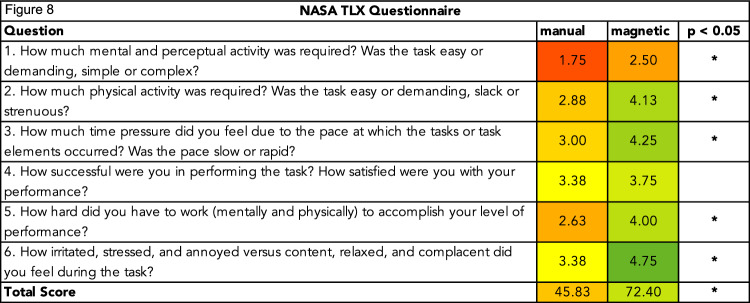
NASA TLX ratings. Each row shows the average rating for the corresponding item on a five-point Likert scale from 1 (worst) to 5 (best) for both navigation methods. For improved readability, scores were inverted for items for which lower values were desirable

The average ratings for each item of the NASA TLX questionnaire are shown in Fig. 8. The mean total score was significantly higher for magnetic navigation, rising from 45.83 ± 16.67 to 72.40 ± 13.54. Magnetic navigation showed statistically significant improvements for items 1, 2, 3, 5, and 6.

### Completion times

**Fig. 9:**
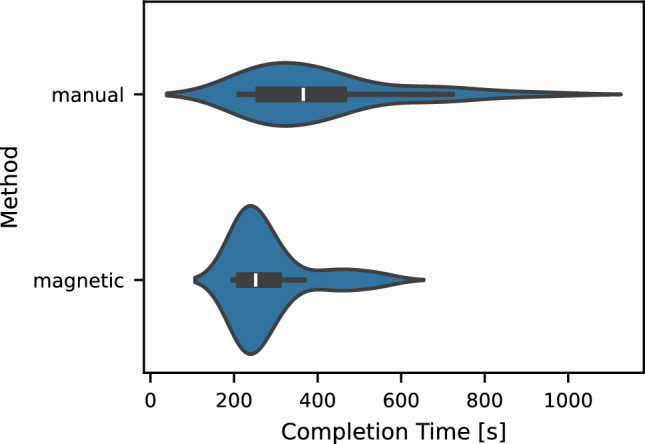
Task completion times. The violin plots show the completion times for all runs performed by the electrophysiologists. The mean completion times for manual and magnetic navigation were 411.0 ± 181.47 s and 284.61 ± 94.63 s, respectively. The box plots inside the violins indicate the median, interquartile range, and data range excluding outliers

In addition to the usability and workload-related aspects, we collected task completion times. The completion times of the electrophysiologists are shown in Fig. 9. Manual and magnetic catheter navigation showed average completion times for a run of 411.0 ± 181.47 s and 284.61 ± 94.63 s, respectively.

**Fig. 10:**
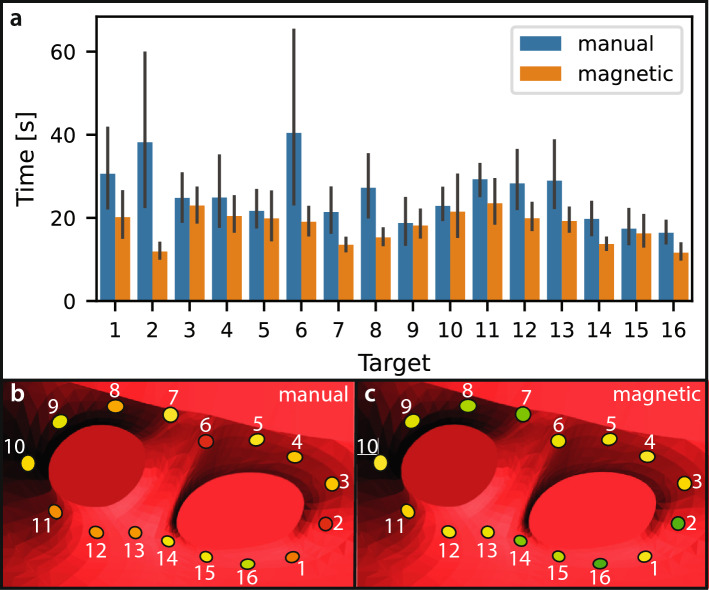
Per-target completion times. **a** Completion times for each target, with error bars indicating the standard deviations. **b** and **c** show the spatial layout of targets and corresponding color-coded completion times for manual and magnetic navigation, respectively. Red indicates longer completion times, while green indicates faster completion

Per-target completion times of the electrophysiologists are presented in Fig. 10. The data reveal a consistent trend: targets that required longer times using manual navigation also tended to be slower with magnetic navigation. Nevertheless, several targets were completed substantially faster with magnetic navigation compared to manual navigation, suggesting potential advantages in navigating to difficult regions.

## Discussion

Previous research has demonstrated that robotics can offer several benefits in the operating room, such as improved dexterity, accuracy, and precision. However, these technical advantages primarily reflect how well a single procedure can be executed. In practice, electrophysiologists often perform several procedures per day, many of which last for several hours. In such contexts, factors like usability and workload become critical when evaluating the overall performance of a system.

The usability of manual and magnetic navigation was assessed using the SUS questionnaire, which captures several aspects related to system usability. While the average SUS score significantly improved with magnetic navigation, not all individual items showed statistically significant improvement. For instance, magnetic navigation received a slightly lower numerical score for item 4, suggesting that technical assistance would still be required to operate the system. Although this aspect may improve over time once operators are more familiar with the system, it suggests that a robotic system technician would still be necessary for seamless operation. In contrast, items 1 and 3 improved significantly with magnetic navigation, indicating that users found the system easy to use and would like to use it more frequently. This emphasizes that the system was well-received by users, despite the need for training and technical support. Importantly, this finding is consistent with the clinical reality: surgeons can prefer a new system but still require technical support, especially since patient safety remains the highest priority.

The workload assessment using the NASA TLX score indicated that magnetic navigation significantly reduced the overall perceived workload. Magnetic navigation outperformed manual navigation in five of the six items. The only item where magnetic navigation did not show a statistically significant advantage was perceived success (item 4). This finding aligns with expectations. Participants were equally successful in completing the tasks using both methods, which explains the lack of difference in perceived success.

For the remaining items—1, 2, 4, 5, and 6—magnetic navigation significantly improved performance, indicating that magnetic navigation allows for more relaxed, physically comfortable, and effortless control of the catheter. This is also reflected in the total NASA TLX score, which significantly and substantially improved using magnetic navigation. The reduced perceived mental workload and stress levels suggest that magnetic navigation can contribute to a healthier work environment for operators, reducing the risks of stress-related malpractices and burnout. Additionally, remote magnetic navigation via a controller allows operators to maintain more neutral and ergonomic positions, as reflected in the improved perceived physical workload scores. This could help prevent these types of occupational injury among surgeons in addition to protecting them from radiation exposure due to its remote nature.

With regard to procedure times, our study shows that magnetic navigation can reduce navigation time in catheter ablation procedures, highlighting its potential for improved navigation efficiency. However, this does not necessarily translate into shorter overall procedure durations, which also include setup, mapping, ablation, and, in some cases, commuting between the operating room and the control room. In fact, the total procedure time is often dominated by the ablation time, which tends to be longer in remote magnetic navigation due to lower contact forces compared with manual ablation [21]. Furthermore, system setup, sterilization, draping, and other preparatory steps may take longer for robotic procedures, particularly in less experienced labs [10], and commuting between the control room and operating table to manually adjust the positioning of catheters can further prolong the procedure [22]. Therefore, while our results suggest greater navigation efficiency with magnetic navigation, shorter overall procedure times will depend on addressing these additional workflow-related factors.

The robotic system’s capability for telesurgery is another interesting aspect. Despite the large physical distance of ca. 1300 km between the robotic system and the participants, magnetic navigation was able to achieve these impressive results. In combination with recent catheter ablation telesurgery studies by Ailoaei et al. [23] and Liu et al. [24], our findings support the technical feasibility and ergonomic potential of magnetic navigation in a telesurgical setting. This could be particularly relevant for expertise sharing and mentoring in difficult cases or interventions in regions with limited local expertise [20]. However, due to the high technical complexity of ablation procedures, telesurgery would not eliminate the need for trained personnel at the patient’s side. Personnel for mapping, electrograms, ablation, and nursing would still require a considerable amount of expertise on site.

Because of the high prevalence of cardiac arrhythmias and the complexity of their treatment, catheter ablation is a rapidly evolving field with frequent technological advances. One recent innovation is pulsed field ablation (PFA), a nonthermal energy source delivered in a single-shot manner that has the potential to significantly simplify catheter navigation in ablation procedures. Current data suggest that PFA may be transformative in atrial fibrillation care [25]. However, long-term efficacy data remain limited [26], and point-by-point thermal ablation continues to be widely used in most centers worldwide. Furthermore, evidence supporting PFA applications beyond pulmonary vein isolation remains sparse [26, 27], suggesting that thermal point-by-point ablation will continue to play a major role in the foreseeable future.

Catheter ablation can be performed with either fixed or steerable sheaths in both navigation methods. A large EHRA survey from 2020 reported that approximately 50% of participating physicians use steerable sheaths for catheter ablation in atrial fibrillation [28]. Remote robotic navigation systems can also be operated with steerable sheaths [29]. Because steerable sheaths are not universally used in clinical practice, in this study we compared magnetic catheter navigation to conventional manual navigation without a steerable sheath to maintain a symmetric setup and to isolate the effect of the navigation modality on workload and usability. Future studies could evaluate usability and workload under configurations that include steerable sheaths to further extend these findings.

This study suggests that magnetic navigation can improve workload and usability. Although similar trends can be expected across various robotic systems, these results are specific to the Navion, the system used in this study. Limitations of the experimental evaluation platform, such as the rigid nature of the heart model, and the absence of blood flow are discussed in detail elsewhere [17]. Furthermore, this study focused on workload and usability during navigation of the ablation catheter. It did not investigate steps such as vascular access, transseptal puncture, placement of diagnostic catheters, cardiac mapping, or tissue ablation.

## Conclusion

In this work, we investigated the performance of the Navion, a mobile eMNS, for catheter ablation. We conducted a user study with eight electrophysiologists, in which each participant completed several in-vitro runs of a navigation task. Participants first performed the experiments using conventional manual catheter navigation, followed by remote magnetic navigation. We collected data on usability, workload, and task completion times. Magnetic navigation significantly improved all three aspects, suggesting that it could bring considerable value to electrophysiology labs. Enhanced usability and reduced workload may benefit not only electrophysiologists, by contributing to mental and physical health, but also clinics and patients by reducing the risk of error or malpractice. The observed reduction in completion times further highlights the potential for more efficient catheter navigation. Overall, these findings support the case for broader adoption of robotic navigation systems in clinical practice.

## Supplementary Information

Below is the link to the electronic supplementary material.

Supplementary file1 (MP4 41390 KB)

Supplementary file2 (MP4 32608 KB)
